# Direct Long Bone Invasion by Basal Cell Carcinoma: A Case Report and Review of the Literature

**Published:** 2013-11-13

**Authors:** Badr M. Al Majed, Stephanie L. Koonce, Dustin L. Eck, Peter Murray, Galen Perdikis

**Affiliations:** ^a^Department of Plastic Surgery, Mayo Clinic, Jacksonville, Fla; ^b^Department of General Surgery, Mayo Clinic, Jacksonville, Fla; ^c^Department of Orthopedics, Mayo Clinic, Jacksonville, Fla

**Keywords:** basal cell carcinoma, bone invasion, long bone invasion, nonmelanoma skin cancer, skin cancer

## Abstract

**Introduction:** Basal cell carcinoma is the most prevalent form of cancer worldwide, usually arising in the head and neck region, which is cured by surgical excision and rarely invades or metastasizes. Many reports exist of bony invasion in the head and neck but very rarely into long bones. **Methods:** We report an unusual case of basal cell carcinoma that despite surgical excision, directly invaded the left humerus. This article also includes a literature review with possible explanations for the occasionally aggressive behavior of basal cell carcinoma. **Results**: This 68-year-old patient underwent wide resection of the affected left upper arm skin, tissue, and diaphyseal segment with clear margins. The defect was reconstructed with a vascularized free fibula bone graft, pedicled latissimus muscle flap, and split-thickness skin graft. **Conclusions:** Long bone invasion by BCC is extremely rare and not well reported. There are more biologic explanations for overtly aggressive behavior that BCC may exhibit such as in this case.

Basal cell carcinoma (BCC) is the most prevalent form of cancer worldwide, accounting for more than 75% of skin cancers in the United States, with a 30% lifetime risk.^1^^,^^2^ Typically they are indolent tumors that most commonly affect elderly fair-skinned males, arise in the head and neck region, and are cured by surgical excision in more than 90% of cases.^1^^,^^3^ Although they are usually slow growing tumors, some tumors may grow rapidly.^4^^,^^5^ Rarely, they may invade adjacent structures.^3^ Metastasis of BCC is extremely uncommon with an estimated rate ranging from 0.0028% to 0.55%. Only around 300 cases of metastatic BCC have been reported since the first reported case by Beadles in 1894.^6^^,^^7^ While bone invasion by BCC is well reported in the head and neck region,^8^^-^12 direct long bone invasion is not. To our knowledge, only 8 cases of BCC with direct long bone invasion have been reported in the literature, and only one of them was in the upper extremity.^2^^,^^5^^,^^8^^,^^13^^-^17 We report a case of basal cell carcinoma with a rare presentation of aggressive invasion of a long bone.

## CASE REPORT

**Figure 1:**
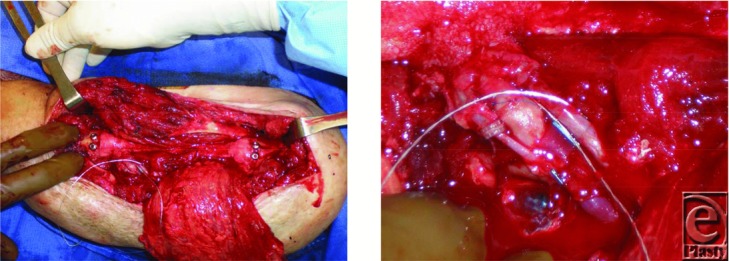
Reconstruction was achieved using a vascularized free fibula bone graft to the left humerus, pedicle latissimus muscle rotational flap, and split-thickness skin graft.

**Figure 2:**
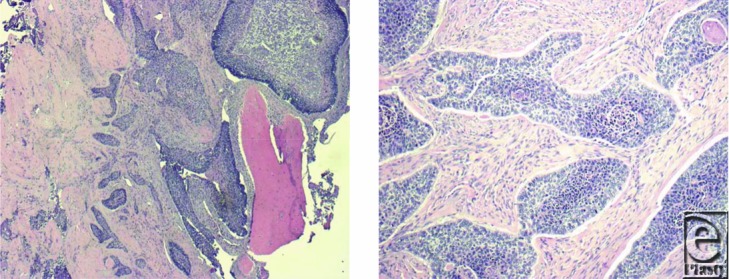
Recurrent BCC with invasion of the humerus.

A 68-year-old male presented to our facility in 2012 with a local recurrence of a left upper arm basal cell carcinoma. His past medical history was otherwise noncontributory. His original tumor was located in the skin of the deltoid region and had developed when the patient was 65 years old. It was treated by wide local excision in 2009. The histopathology report confirmed a diagnosis of BCC with the tumor appearing completely excised. One year later, he experienced a local recurrence of the lesion, and this was treated with further wide local excision to negative margins. Three years after initial presentation, the patient presented to our institution for evaluation of recurrent disease secondary to a concern over skin changes. Magnetic resonance imaging and computed tomography demonstrated recurrent tumor with invasion of the left upper humerus. Computed tomography–guided biopsy of the humerus demonstrated BCC. The patient underwent a wide resection of the skin, soft tissues, and the diaphyseal segment of the humerus. Reconstruction was achieved using a vascularized free fibula bone graft to the left humerus, a pedicle latissimus muscle rotational flap, and a split-thickness skin graft (Fig 1). The patient made an uneventful recovery and was discharged on postoperative day 3. Final pathology revealed an infiltrating BCC, forming a soft tissue mass with invasion of adjacent humeral bone (Fig 2).

**Figure 3:**
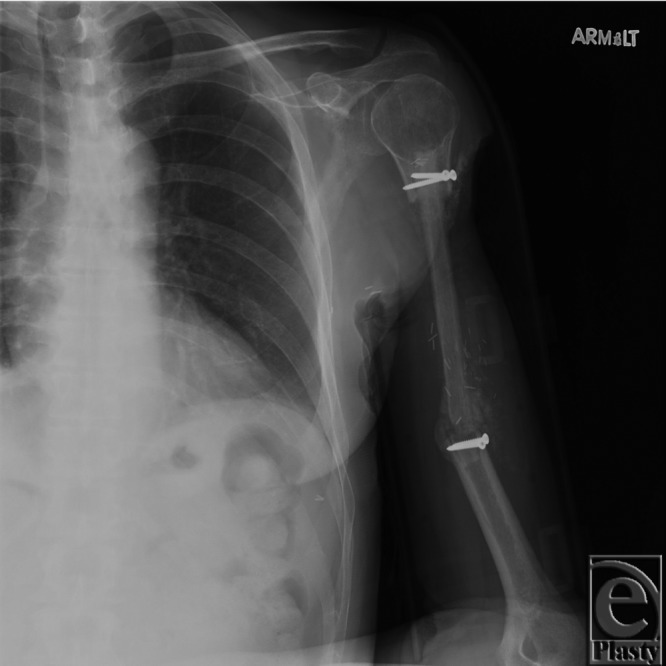
Fracture at graft site.

At 4 months, the patient experienced a fracture of the ipsilateral distal humerus at the graft site that was conservatively treated with a Sarmiento brace (Fig 3). At the last follow-up 6 months after surgery, the patient had no evidence of disease recurrence and a full range of motion of his left arm.

## DISCUSSION

Basal cell carcinoma is usually a cancer that patients would die with rather than from, as it is mostly a local slow-growing tumor that rarely metastasizes or invades adjacent structures.^3^ Nevertheless, once metastasis is diagnosed, the prognosis is poor, with median survival length of 8 months after the first sign of metastasis^18^ and 5-year survival rates as low as 10%.^7^ However, there are some extreme cases who have survived significantly longer.^19^^,^^20^

Basal cell carcinoma is mostly clinically diagnosed. To enhance the accuracy of the diagnosis, or when doubt exists, biopsy is indicated for histological confirmation. Radiological imaging, like computerized tomography, may be used for assessment and management planning when invasion or metastasis is suspected. Tumor size, site, margins, histological subtype and aggression, failure of previous treatment, and immunosuppression are factors that have been identified to affect the risk level of recurrence and the prognosis of the lesion.^21^ Depending on these factors, the treatment choice should be individualized for each patient. There are multiple modalities for BCC treatment. Currently, surgical excision is the standard of care for BCC.^21^^,^^22^ Mohs surgery is preferred where there is lack of tissue such as eyelid or nasal tip. Other options that do not allow histological confirmation of tumor clearance include cryosurgery and curettage. Nonsurgical options include photodynamic therapy, laser therapy, radiotherapy, local treatments like imiquimod and 5-fluorouracil, and a new systemic drug named *Vismodegib*.^21^^-^24 A recent study comparing different nonsurgical treatment modalities showed that imiquimod had the highest efficacy while photodynamic therapy had the highest recurrence rate.^25^ The Food and Drug Administration recently approved a selective Hedgehog (Hh) signaling pathway inhibitor oral drug called *Vismodegib* that can be included in the treatment regimen for cases with BCC that cannot be treated with surgery, that have relapsed after surgery, or that have metastasized.^24^ This novel drug targets the malfunctioning Hh signaling pathway, a key regulator of the proper development of the embryonic and adult cells, which is implicated in the development and progression of several types of cancer, including BCC.^5^ Although the limited early data are promising, more than half of the patients who used *Vismodegib* for nonmelanoma skin cancer discontinued its use at 18 months of treatment for intolerable side effects.^26^^,^^27^

Adjuvant therapy has generally been radiotherapy. However, the treatment of invasive, recurrent, and large primary tumors is more complex. These types of tumors, such as this report, require confirmation of diagnosis, imaging, and a multidisciplinary treatment approach with surgery remains the mainstay of the treatment plan.

On the molecular level, normally a cell membrane receptor called Patched tonically inhibits another cell membrane protein called Smoothened (Smo). The activation of Smo, which is achieved by the binding of the Sonic Hedgehog to Patched, induces the production of a number of proteins. Genetic mutations to the gene responsible for the production of Patched, which were identified in patients with both sporadic and hereditary BCC, lead to the loss of the inhibitory effect exerted on Smo by Patched, which in turn leads to upregulation of the end products of the Hh/PTCH/Smo pathway.^5^^,^^28^ TGF-β, 1 of the 2 classes of TGF and an end product of the Hh pathway, normally inhibits proliferation and promotes differentiation of the epithelial cells. Although its role in BCC is not fully understood, it has been suggested that TGF-β increases the proliferation and promotes Epithelial-Mesenchymal transition (EMT) of the tumor cells, a process that will be discussed later in this article, and it also inhibits apoptosis of the surrounding active stroma, resulting in BCC progression.^29^^,^^30^

Studies have tried to explain the occasional atypical aggressive behavior of BCC and the reasons behind the tendency to invade and metastasize in rare instances. Abnormalities in several biological processes, such as apoptosis, cell adhesion, or their components, have been implicated in different studies as possible explanations for such behavior. Apoptosis, or programmed cell death, is a process by which the abnormal cells are sentenced to death.^31^ Some of the proteins playing essential roles in the different steps of the apoptosis, such as p53, Bcl-2, and Fas, have been found to be dysregulated in aggressive cases of invasive BCC.^29^

Epithelial-Mesenchymal transition is a type of epithelial plasticity that is characterized by transdifferentiation of epithelial cells toward a mesenchymal cell type. Tumor invasion has many similarities to EMT, such as loss of adhesion within the tissue, decreased E-cadherin expression, which plays a key role in the cell-to-cell adhesion, and increased cell motility.^32^ A study conducted by Majima et al^33^ revealed a significant downregulation of E-cadherin in invasive and metastatic BCC compared with noninvasive nodular BCC. Research conducted by Marsh et al showed a direct relationship between aggressive BCC and αvβ6 Integrin, a transmembrane receptor that mediates cell adhesion and that is capable of promoting both invasion and fibrosis. αvβ6 was found to be overexpressed in the invasive histologic types of BCC, unlike the low-risk nodular variant.^34^ Other molecules like β-catenin and CD44 were found to be upregulated in the tumor margins and the surrounding stroma of invasive BCC; thus, it may influence the migration of tumor cells and progression of the disease.^5^^,^^35^

Early recognition and excision of primary BCC with careful follow-up mostly prevents possible unfortunate outcomes and helps to lower the burden that the progression of the tumor might cause. However, we should emphasize that BCC can grow rapidly and can become aggressive, severely disfiguring, and can invade or metastasize with or without patient neglect. There are many interesting findings in studies looking at BCC with aggressive behavior, but further research is still required to better understand the pathophysiology of BCC and how to recognize which tumors may display aggressive behavior. The treatment of these aggressive tumors needs to be methodical and similarly aggressive, with surgery remaining the mainstay of treatment.
